# Alcohol consumption is associated with lower self-reported disease activity and better health-related quality of life in female rheumatoid arthritis patients in Sweden: data from BARFOT, a multicenter study on early RA

**DOI:** 10.1186/1471-2474-14-218

**Published:** 2013-07-24

**Authors:** Stefan Bergman, Sofia Symeonidou, Maria L Andersson, Maria K Söderlin

**Affiliations:** 1 Department of Clinical Sciences, Lund, Section of Rheumatology, Lund University, Lund, Sweden; 2 Research and Development Center, Spenshult Rheumatology Hospital, Oskarström SE-313 92, Sweden

**Keywords:** Rheumatoid arthritis, Alcohol, EULAR response criteria, Health-related quality of life

## Abstract

**Background:**

Earlier studies have found a positive effect of alcohol consumption, with a reduced disease activity in rheumatoid arthritis (RA). The aim of this study was to assess alcohol consumption and its association with disease activity and health related quality of life (HRQL) in Swedish RA patients.

**Methods:**

Between 1992 and 2005, 2,800 adult patients were included in the BARFOT study of early RA in Sweden. In 2010 a self-completion postal questionnaire was sent to all 2,102 prevalent patients in the BARFOT study enquiring about disease severity, HRQL, and lifestyle factors. Alcohol consumption was assessed using the validated AUDIT-C questionnaire.

**Results:**

A total of 1,238 out of 1,460 patients answering the questionnaire had data on alcohol consumption: 11% were non-drinkers, 67% had a non-hazardous drinking, and 21% were classified as hazardous drinkers. Women who drank alcohol reported lower disease activity and better HRQL, but there were no association between alcohol consumption and disease activity in men. For current smokers, alcohol use was only associated with fewer patient-reported swollen joints. The outcome was not affected by kind of alcohol consumed.

**Conclusions:**

There was an association between alcohol consumption and both lower self-reported disease activity and higher HRQL in female, but not in male, RA patients.

## Background

Alcohol has been shown to have immunosuppressive effects in mouse models [1]. Two large studies from the UK and Switzerland on rheumatoid arthritis (RA) patients have reported positive effects of moderate drinking, with a reduced disease activity and a better outcome in RA [2,3]. The Swiss study showed that alcohol had significant positive effects with less radiographic progress in men, but not in women [3]. Drinking has also been reported to be associated with lower risk of developing RA [2,4,5]. It is not known whether the kind of alcohol has any influence on disease activity in RA.

Little is known about alcohol consumption and its association with disease activity in Swedish RA patients. The aim of this study was to assess alcohol consumption and its association with disease activity and health related quality of life (HRQL) in a large longitudinal observational study of Swedish RA patients. Our hypothesis was that moderate drinking would be associated with better HRQL and less pain. We also hypothesized that RA patients who drank heavily would have poorer HRQL and function. A second aim was to determine whether there were any gender differences regarding the effect of alcohol on disease activity, and whether the kind of alcohol the patients drank was associated with disease activity. We hypothesized that wine drinkers would have a particularly good outcome.

## Methods

During the period 1992–2005, 2,800 patients over 18 years of age were enrolled in the BARFOT (Better Anti-Rheumatic FarmacOTherapy) study, a multicenter longitudinal observational study of patients with early RA in southern Sweden [6-8]. All patients fulfilled the American College of Rheumatology RA classification criteria from 1987 [9] and in this study all patients had a disease duration of ≤ 2 years. Disease activity was evaluated at inclusion, at 3, 6, and 12 months, and at 2, 5, 8, and 15 years. Treatment with disease-modifying anti-rheumatic drugs (DMARDs), glucocorticoids and biologics was registered at all follow-up points. Between March and September 2010, all patients who were still alive in the BARFOT study (n = 2,102) received a self-completion postal questionnaire with visual analog scales (VAS) for general health and pain, the Swedish version of the Health Assessment Questionnaire (HAQ) [10,11], patient-reported swollen joint counts (SJCs) and tender joint counts (TJCs) (of 28), assessment of lifestyle factors such as smoking and alcohol consumption, and current and past medication with DMARDs, glucocorticoids, and biologics. Health-related quality of life (HRQL) was assessed using the EQ-5D (EuroQol). Part 1 of the EuroQol questionnaire assesses self-reported problems involving 5 domains: mobility, self-care, usual activities, pain/discomfort, and anxiety/depression. Each domain is divided into 3 levels of severity: no problem, some problems, and extreme problems. A time-tradeoff procedure in a normal adult population (with 3,395 respondents) in the UK has been used to obtain EuroQol utility weights that vary between 1 and −1 [12-16].

Drinking was assessed with the AUDIT-C [17-20], which is a validated subset of the consumption items of the full AUDIT (Alcohol Use Disorder Investigation Test) [21-26]. The AUDIT-C has been shown to outperform the full AUDIT instrument when measuring high-volume drinking [21,27].

The AUDIT-C questionnaire consists of three questions, giving a total score of 0 to 12 points:

(1) How often do you have a drink containing alcohol? Never (0 points), Once a month or even less often (1 point), 2–4 times a month (2 points), 2–3 times a week (3 points), and 4 times a week or more (4 points).

(2) How many standard drinks containing alcohol do you have on a typical day? 1-2 (0 points), 3–4 (1 point), 5–6 (2 points), 7–9 (3 points), and 10 or more (4 points).

(3) How often do you have six or more drinks on one occasion? Never (0 points), Less often than once a month (1 point), Every month (2 points), Every week (3 points), and Daily or almost daily (4 points).

The Swedish National Institute of Public Health has set limits for hazardous drinking at AUDIT-C ≥ 4 points for women and ≥ 5 points for men (http://www.fhi.se and personal communication). In this study, these patients were labelled hazardous drinkers. Non-hazardous drinkers in this study were patients who did drink alcohol, but who had less total points in the AUDIT-C than hazardous drinkers.

Patients were also asked to tick in a box “What do you prefer to drink when you drink alcohol? (beer/wine/spirits)”. Some patients ticked more than one box, and these patients were classified as “mixed” drinkers. The postal questionnaire in 2010 had a picture associated with these questions showing that one “drink” of alcohol corresponds to 500 ml of light beer, 330 ml of strong beer, 100–150 ml of red or white wine, 50–80 ml of fortified wine (e.g. sherry), or 40 ml of spirits, e.g. whisky.

Smoking status was recorded at the time of the postal questionnaire in 2010 (never-smokers, current smokers, and previous smokers). Occupation was also recorded and coded according to the latest version of the Categorization of Socioeconomic Status (Socioekonomisk indelning, SEI) in Sweden. The socioeconomic status of workers was classified as blue-collar, lower white-collar, upper white-collar, self-employed, and other occupation according to the SEI.

Altogether, two reminders were sent to the patients who did not respond to the first and second mailing of the self-completed questionnaire in 2010. All patients received written information about the questionnaire in 2010, and the Ethical Committee of Lund University approved the BARFOT study and the self-completed postal questionnaire in 2010.

### Statistics

Statistical analyses were performed using SPSS 18.0 statistical software. All significance tests were two-tailed and were conducted at the 5% significance level. To test differences between groups, Mann–Whitney test or the independent t-test was used for continuous variables and the chi-square test was used for proportions. For non-parametric values, Kruskall-Wallis test was used to analyze differences between the different drinking categories. Pairwise Kruskall-Wallis post hoc analyses were performed to determine which variables differed significantly from each other, stratified for the different drinking categories. Post hoc analyses using standardized residuals were used to assess the differences in DMARD treatment and differences between sexes, stratified according to alcohol consumption. Multiple logistic regression analyses were used to determine whether alcohol consumption was an independent predictive factor for a score over the median value of HAQ, EuroQol, and VAS global health in the 2010 questionnaire. The following variables were included in the regression analyses at the time of the 2010 questionnaire: drinking (none, non-hazardous, hazardous), age, sex, disease duration, smoking (no, previous, current), SEI class (blue-collar, lower white-collar, upper white-collar, self-employed, other), and number of previous DMARDs and biologics. There was no colinearity in the variables entered into the multiple logistic regression analyses.

## Results

Altogether, 1,460 patients who had answered the 2010 self-completion postal questionnaire and who were > 18 years of age and had a disease duration of ≤ 24 months were included in this study. The patient-reported disease activity and demographics from the postal questionnaire in 2010 are shown in Table 1.

**Table 1:** **Demographics and disease activity variables in the questionnaire in 2010**^**a**^

Answered the 2010 postal questionnaire	n = 1,460
Age in years, mean (SD)	65 (13)
Percentage females	70
HAQ	0.50 (0–1.0)
VAS global, mm	30 (10–50)
VAS pain, mm	30 (10–50)
Number of swollen joints (of 28)	1 (0–5)
Number of tender joints (of 28)	3 (1–8)
EuroQol	0.73 (0.69–0.80)
Number of current DMARDs and biologics	1.0 (1.0–2.0)
Number of previous DMARDs and biologics	1.0 (1.0–2.0)
Current methotrexate, %	60
Current glucocorticoids, %	23
Smokers, %	17

^a^Unless otherwise stated, data are median (interquartile range).

*HAQ* Health Assessment Questionnaire, *VAS* visual analog scale, *DMARDs* disease-modifying anti-rheumatic drugs.

Patients who did not answer the 2010 questionnaire (579/2104, 27.5%) had higher mean DAS28 (5.4 for non-responders vs. 5.2 for responders, p = 0.01), higher mean VAS health (48 mm vs. 45 mm, p = 0.008), higher mean number of SJCs (11 vs. 10, p = 0.03) at inclusion, were more often smokers (30% vs. 24%, p = 0.01) and were less often RF-positive (58% vs. 63%, p = 0.02) compared to the patients who had answered the 2010 questionnaire.

Alcohol consumption data at the time of the 2010 questionnaire were available for 1,238/1,460 (85%) patients. The 222 patients (15%) who did not answer the alcohol questions were older, with a mean age at inclusion was 59 years for those who did not answer vs. 54 years for those who answered (p = 0.0001), had higher mean ESR (38 mm vs. 34 mm, p = 0.03), and were more often men (37% vs. 29%, p = 0.02). There were no significant differences in DMARD treatment or glucocorticoid treatment at baseline, or in rheumatoid factor status or smoking status at baseline or in 2010, between those who answered the alcohol-related questions and those who did not.

In the entire cohort of 1,238 patients, 140 (11%) were non-drinkers, 834 (67%) had a non-hazardous drinking, and 264 (21%) were classified as hazardous drinkers. Significantly more women were non-drinkers than men (14% vs. 4%, p = 0.0001). Significantly more blue-collar workers were hazardous drinkers (standardized residual −2.1). Eight per cent of the patients who were non-drinkers were current smokers, as compared to 17% of non-hazardous drinkers and 21% of hazardous drinkers (p = 0.0001). Of the patients ≤ 30 years of age, 39% drank heavily as compared to 23% in the 31- to 65-year age group and 12% in patients > 65 years. Older patients were more often non-drinkers (overall p = 0.0001).

### Disease activity data stratified for drinking and sex

Disease activity was stratified according to drinking and sex (Table 2). Non-hazardous and hazardous drinkers reported lower disease activity and higher EuroQol scores, with the exception of SJCs. A lower proportion of hazardous drinkers had undergone glucocorticoid treatment.

**Table 2:** **Disease activity stratified for alcohol and sex**^**a**^

	**All respondents**	**No alcohol consumption**	**Non-hazardous drinking**	**Hazardous drinking**	
	**n = 1,238**	**n = 140**	**n = 834**	**n = 264**	**p**^**b**^
Number of tender joints (of 28)	3 (1–8)	4 (1–11)	3 (1–8)	3 (1–7)	0.02
Men	2 (0–6)	2 (0–5)	2 (0–6)	2 (0–5.25)	0.94
Women	4 (1–10)	5 (2–12)	4 (1–9)	3 (1–7.25)	0.03
Number of swollen joints (of 28)	1 (0–5)	2 (0–6)	2 (0–5)	1 (0–4)	0.11
Men	0 (0–3)	0 (0–1)	0 (0–4)	1 (0–3)	0.31
Women	2 (0–6)	2 (0–6)	2 (0–6)	1 (0–4)	0.05
VAS global, mm	30 (10–50)	40 (20–50)	30 (10–50)	20 (10–40)	0.0001
Men	20 (10–50)	20 (10–45)	20 (10–40)	20 (10–40)	0.69
Women	30 (10–50)	40 (20–60)	30 (20–50)	20 (10–40)	0.0001
VAS pain, mm	30 (10–50)	40 (20–60)	30 (10–50)	30 (10–50)	0.001
Men	30 (10–50)	30 (20–40)	20 (10–40)	20 (0–40)	0.18
Women	40 (20–60)	40 (20–60)	30 (20–50)	30 (10–60)	0.03
EuroQol	0.76 (0.69–0.80)	0.73 (0.62–0.80)	0.76 (0.69–0.80)	0.80 (0.73–0.80)	0.0001
Men	0.80 (0.73–0.85)	0.73 (0.67–0.80)	0.80 (0.73–0.85)	0.80 (0.73–1.0)	0.07
Women	0.73 (0.66–0.80)	0.73 (0.59–0.80)	0.73 (0.69–0.80)	0.80 (0.73–0.80)	0.005
HAQ	0.38 (0–0.90)	0.75 (0.25–1.38)	0.5 (0–0.88)	0.25 (0–0.75)	0.0001
Men	0.25 (0–0.75)	0.13 (0–0.44)	0.25 (0–0.63)	0.13 (0–0.75)	0.80
Women	0.63 (0.13–1.239	0.88 (0.34–1.38)	0.50 (0.13–1.0)	0.38 (0–0.75)	0.0001
Number of current DMARDs and biologics, mean (SD)	1.1 (0.8)	0.9 (0.9)	1.1 (0.7)	1.2 (0.8)	0.03
Men	1.0 (0.7)	1.1 (0.7)	1.0 (0.7)	1.2 (0.7)	0.30
Women	1.1 (0.8)	0.9 (0.9)	1.1 (0.7)	1.1 (0.9)	0.02
Number of previous DMARDs and biologics, mean (SD)	1.7 (1.4)	1.5 (1.2)	1.8 (1.5)	1.7 (1.3)	0.18
Men	1.0 (0.7)	1.3 (0.5)	1.5 (1.3)	1.6 (1.1)	0.24
Women	1.8 (1.5)	1.5 (1.2)	1.9 (1.6)	1.8 (1.3)	0.03

^a^Unless otherwise stated, data are median (interquartile range).

^b^The p-values relate to the different drinking categories.

*HAQ* Health Assessment Questionnaire, *VAS* visual analog scale, *DMARDs* disease-modifying anti-rheumatic drugs.

Women scored worse than men in all of the disease activity variables and EuroQol (p = 0.0001 for all). Women were also generally younger than men (64 vs. 68; p = 0.0001). Women had had significantly more DMARDs previously than men (1.49 vs. 1.22, p = 0.0001), and had had more previous treatment with biologics (0.3 vs. 0.2, p = 0.004). There were no significant differences in the mean number of current DMARDs, stratified according to gender (p = 0.61).

For women, there were significant associations between the drinking categories and all of the disease activity variables studied. However, no such differences were seen for men (Table 2).

### Stratification for smoking

Current smokers only showed a significant association between drinking and the number of SJCs (p = 0.02), where the difference was between hazardous drinkers and non-drinkers. Previous smokers had significant associations between drinking and VAS global (p = 0.01), VAS pain (p = 0.03), and HAQ (p = 0.0001), the differences for the VAS scales being between hazardous drinkers and non-drinkers, and for HAQ between all of the drinking groups. For non-smokers, drinking was associated with lower disease activity in all of the variables studied with the exception of SJCs (p = 0.33; the rest of the data not shown).

### Treatment with DMARDs and glucocorticoids

There were significant differences in DMARD and glucocorticoid treatment, stratified according to alcohol consumption. At 6 months of follow-up, 19% of the non-drinkers had not received any DMARD treatment, as opposed to 8% of hazardous drinkers. At 2 years of follow-up, 25% of the non-drinkers had not received DMARDs, as opposed to14% of the hazardous drinkers. Hazardous drinkers more often received combination treatment: 18% vs. 7% for non-drinkers. At 5 years of follow-up, 31% of non-drinkers had not received any DMARDs, as opposed to 15% of the hazardous drinkers. Hazardous drinkers more often received biologics: 21% vs. 11%. At 8 years of follow-up, 39% of non-drinkers had not received DMARDs, as opposed to18% of hazardous drinkers. Hazardous drinkers more often received biologics: 26% vs. 13%. Thus, non-drinkers received DMARDs, biologics, and combination treatment less often than hazardous drinkers.

There was no significant difference in glucocorticoid treatment at any time point, stratified according to alcohol consumption. There was no association between previous or current use of methotrexate and drinking (p = 0.92 and p = 0.13, respectively).

### Multiple logistic regression analyses

Drinking at the time of the 2010 questionnaire was not independently associated to a better outcome in VAS global, EuroQol, or HAQ (Table 3). However, when socioeconomic class and previous treatment with DMARDs and biologics were omitted in the regression analyses, hazardous alcohol consumption was associated with better EuroQol outcome (OR = 1.67, 95% CI = 1.02–2.75, p = 0.04), better HAQ outcome (OR = 0.53, 95% CI = 0.32–0.86, p = 0.01), and better VAS global outcome (OR = 0.60, 95% CI = 0.37–0.98, p = 0.04). Current smoking emerged as an independent negative prognostic factor, but only when using EuroQol as outcome, and only when omitting socioeconomic class and previous anti-rheumatic treatment from the model (data not shown).

**Table 3:** Odds ratios (OR) and 95% confidence intervals (95% CI) for multivariable analyses of factors potentially associated to VAS global, EuroQol and HAQ

		**VAS global**	**EuroQol**	**HAQ**
		**OR (95% CI)**	**OR (95% CI)**	**OR (95% CI)**
Sex	Women	1.0	1.0	1.0
	Men	0.59 (0.43–0.82)	2.09 (1.50–2.93)	0.45 (0.32–0.63)
Age		1.02 (1.01–1.04)	0.98 (0.97–0.99)	1.04 (1.03–1.06)
Disease duration		1.04 (1.01–1.08)	1.00 (0.96–1.04)	1.06 (1.02–1.10)
Alcohol use				
	None	1.0	1.0	1.0
	Non-Hazardous	0.72 (0.43–1.20)	1.24 (0.74–2.07)	0.92 (0.55–1.53)
	Hazardous	0.64 (0.36–1.16)	1.48 (0.82–2.68)	0.70 (0.38–1.26)
Socio-economic group				
Blue collar		1.0	1.0	1.0
Lower white collar		0.69 (0.51–0.92)	1.54 (1.14–2.08)	0.81 (0.60–1.10)
Upper white collar		0.44 (0.25–0.80)	1.56 (0.88–2.79)	0.64 (0.36–1.16)
Self-employed		2.58 (0.51–13.10)	5.81 (0.69–48.74)	0.35 (0.07–1.79)
Others		0.88 (0.31–2.47)	1.04 (0.34–3.12)	0.54 (0.18–1.66)
Smoking	Never	1.0	1.0	1.0
	Previous	0.79 (0.57–1.08)	0.95 (0.68–1.32)	1.02 (0.73–1.42)
	Current	1.06 (0.70–1.61)	0.58 (0.38–0.88)	1.46 (0.95–2.26)
No. DMARDs or biologics		1.15 (1.04–1.28)	0.71 (0.63–0.79)	1.51 (1.34–1.71)

### Kind of alcohol and disease activity

In the 2010 postal self-completion questionnaire, the patients were asked what kind of alcohol they preferred to drink. Data for this were available for 1,218/1,460 (83%) of the patients. Two hundred and six (16.9%) preferred to drink beer, 791 (64.9%) preferred to drink wine, and 76 (6.2%) preferred to drink spirits. A total of 145 (11.9%) drank mixed kinds of alcohol. Of the patients who drank beer, 17% were hazardous drinkers; of the patients who drank wine, 25% were hazardous drinkers; of the drinkers of spirits, 18% drank hazardously; and of those who drank different kinds of alcohol, 33% were hazardous drinkers (p = 0.009). Men preferred to drink beer (31%), wine (37%), spirits (11%), or more than one kind of alcohol (21%) whereas women preferred to drink wine (78%), but not beer (10%), spirits (4%), or more than one kind (8%) (p = 0.0001). There was no significant association between the kind of alcohol and the disease activity variables in the 2010 postal questionnaire (EuroQol, p = 0.96; HAQ, p = 0.33; TJC, p = 0.65; SJC, p = 0.49; VAS global, p = 0.76; VAS pain, p = 0.71). This did not change when stratified for gender or the quantity of alcohol consumed.

## Discussion

This is the first Swedish study to find an association between drinking and disease activity in RA. Drinking was assessed by the AUDIT-C, a validated self-completion questionnaire, in a cross-sectional manner as part of a postal questionnaire that also assessed patient-derived data on disease activity, HAQ, and HRQL in 2010. We found that there was a significant association between non-hazardous and hazardous drinking and both lower patient-reported disease activity and higher HRQL, confirming one of our hypotheses. However, this effect was only seen in women, a result that we find very surprising. We could not confirm our second hypothesis, that hazardous drinkers have poor outcome. On the contrary, women who drank most reported lowest disease activity and highest HRQL.

It is important to note that we were unable to assess cause and effect regarding alcohol consumption and disease activity in our cross-sectional setting. It is possible that women who had less joint inflammation and better health allowed themselves to drink more. There may also be gender differences in how women and men abstain from drinking because of their RA; for example we were able to show that women were more often non-drinkers than men. We could also show that non-drinkers less often received DMARDs, biologics, and combination treatment than hazardous drinkers. This may reflect the fact that these patients might have had more comorbidities and/or that it was not possible to use anti-rheumatic medication due interactions with other medications, factors that we could not adjust for. It is also possible that some non-drinking patients chose not to use anti-rheumatic medications. Methotrexate treatment was not associated with drinking in our study. Men drank spirits and beer more often than women, who preferred wine, and in men this may possibly have been associated with hazardous drinking during weekends, which we could not control for in this study. There may also be physiological differences in how men and women react to alcohol. Women had higher disease activity, as reported earlier from this same material [28,29], which could affect treatment decisions and drinking patterns.

Drinking was not independently associated to a better outcome in our regression models. However, when socioconomic status and previous anti-rheumatic treatment were omitted from the regression models, hazardous drinking was associated with better global health, HAQ, and EuroQol. There is most likely an interaction between socioeconomic factors and alcohol.

We found that 20% of the patients were hazardous drinkers. The real number of hazardous drinkers (running the risk of having psychosocial problems and organ damage) was most likely even higher. Self-reporting of alcohol consumption is known to give an underestimate of drinking [21].

Alcohol is known as an immunosuppressant in both mice [1] and humans [30]. Heavy drinking has been associated with elevated levels of TNF-α, IL-1, and IL-6 [31], whereas intake of low to moderate amounts of alcohol over a longer time period (years) has been shown to reduce levels of TNF-α, IL-1, and CRP [32,33]. Alcohol also has anti-nociceptive effects [34]. There is thus a theoretical background for association of alcohol consumption with less inflammation in RA.

RA patients who smoked did not show any association between drinking and disease activity, with the exception of lower SJCs. This was also an unexpected finding. We have previously reported, in this same cohort [29,35] and also in RA patients treated with anti-TNF [36], that RA patients who smoke have poorer prognosis. Other studies have confirmed this [37-44]. Current smoking also emerged as an independent negative prognostic factor in this study, but only for the outcome EuroQol, and only when omitting socioeconomic class and previous anti-rheumatic treatment from the models. It is possible that smoking causes more inflammation, or that smoking interacts with the mechanisms driving inflammation, and that alcohol as an immunosuppressant is not able to suppress this. Lifestyle factors may also be involved. Smoking and drinking may also be linked to poorer drug adherence and poorer compliance.

We could see no association between the kind of alcohol consumed and disease activity or HRQL, and could thus not confirm our third hypothesis that patients who preferred to drink wine would do better. There is presently no consensus in the literature on the effects of kind of alcohol consumed on health [45].

To what extent can our results in Sweden be generalized? The Swedish multicenter EIRA case–control study on RA reported that a lower risk of developing RA was associated with alcohol consumption [5]. The patients in that study drank a mean of 2.9 drinks per week [5]. The EIRA study reported that 15% were non-drinkers, 56% were low consumers, 14% were moderate consumers, and 14% were high consumers, figures that are comparable to ours.

How generalizable is this study internationally? A large Swiss longitudinal observational study involving 2,903 RA patients found a trend of reduced radiographic progression in drinkers, specifically in occasional drinkers and those who consumed alcohol on a daily basis, as compared to non-drinkers. There were gender differences. Men who drank alcohol showed a more pronounced reduction in radiographic progression than men who did not drink, whereas in women drinking had no significant influence on radiographic outcome [3]. Thirty-seven per cent of patients in the Swiss study were non-drinkers, which is considerably more than the 11% in our study. Of the patients in the Swiss study who drank alcohol, 82% were occasional drinkers, 15% were daily drinkers, and 3% were heavy drinkers. HAQ at baseline was higher in the Swiss study (1.0–1.3 in that study, as compared to 0.5 in our study) and more patients were on glucocorticoids (about 50% in the Swiss study as compared to 23% in our study) but a comparable number of patients were on methotrexate (61%) [3].

A study from the UK on 873 RA patients and 1,004 controls showed that the risk of developing RA decreased according to the level of alcohol consumption, and that measures of disease activity such as CRP, DAS28, VAS pain, mHAQ (modified HAQ), and Larsen score were inversely associated with increasing frequency of alcohol consumption in both men and women [2]. In the UK study, a higher percentage of patients (37%) than in our study (11%) were non-drinkers, 33% drank 1–5 days a month, 15% drank 6–10 days a month, and 16% drank > 10 days a month. Mean range of HAQ was 0.75–1.0, which was higher than in our study, and that for DAS28CRP was 4.0–4.3 [2]. Our study differs from these earlier studies in that the association between drinking and disease activity and HRQL was evident only in women.

It is a limitation that we do not have any physician derived measurement of disease activity and have to rely on patient reports from the questionnaire, where VAS global and HAQ could represent other aspects of the disease than an active inflammation. We also use patient-derived joint counts. A recent study has assessed the correlations between physician- and patient-derived joint counts in 47 RA patients treated with adalimumab. It showed that the correlation was good for the TJCs and SJCs at baseline (r = 0.87 and r = 0.75) and at 3 months for the TJCs (r = 0.87), but it was somewhat less strong for SJCs (r = 0.58) [46]. A poor correlation between physician- and patient-derived data was found in another study (particularly for low SJCs in the hands) in 447 RA patients using etanercept (r = 0.08–0.55) but there was a stronger correlation for TJCs (r = 0.78) [47]. Selection bias may also be an issue, but 69% of eligible patients responded to the questionnaire. There were small but statistically significant differences at inclusion between those who answered the questionnaire and those who did not. These differences are not likely to change the main findings of this study.

The major strengths of the present study were that we had a large, well-documented patient material and that we used a validated instrument to measure drinking (AUDIT-C). We had a high response rate for both the 2010 questionnaire as a whole (73%) and for the alcohol questions (85%). Our data showed that patients who did not answer the questionnaire had higher disease activity and were more often men, confirming earlier studies on attrition and non-response to questionnaires [48-50]. The patients who died or who did not answer the questionnaire may also have consumed more alcohol.

## Conclusions

There was an association between alcohol consumption and both lower self-reported disease activity and higher HRQL in female, but not male, RA patients in Sweden. For current smokers, alcohol consumption was only associated with fewer swollen joints. We could not see any effect of the kind of alcohol consumed on disease activity. Twenty-one per cent of the RA patients in this study drank hazardously, a figure that is probably an underestimate, and which we find alarming.

## Competing interests

Dr. S. Bergman has received speaking fees for educational events from Pfizer (<$5000). Dr. M. Söderlin has received speaking fees for educational events from Abbott (< $3,000) and MSD (< $1,000), and speaking fees and consultation fees from Pfizer (< $5,000).

## Authors’ contributions

SB, SS, and MS performed the statistical analyses. SB, MA, and MS planned the study, participated in its design and coordination, and reviewed the manuscript. All authors read and approved the final manuscript.

## Authors’ information

Members of the BARFOT study group

Sofia Ajeganova, Maria Andersson, Valentina Bala, Stefan Bergman, Kristina Forslind, Ingiäld Hafström, Catharina Keller, Ido Leden, Bengt Lindell, Ingemar Petersson, Christoffer Schaufelberger, Björn Svensson, Maria Söderlin, Annika Teleman, Jan Theander, and Anneli Östenson.

## Pre-publication history

The pre-publication history for this paper can be accessed here:

http://www.biomedcentral.com/1471-2474/14/218/prepub
